# A giant, complex fronto-ethmoidal ivory osteoma: Surgical technique in a resource-limited practice

**DOI:** 10.4103/2152-7806.74489

**Published:** 2010-12-31

**Authors:** Amos Olufemi Adeleye

**Affiliations:** Skull Base Surgery Unit, Division of Neurological Surgery, Department of Surgery, College of Medicine, University of Ibadan, Ibadan, Nigeria

**Keywords:** Giant fronto-ethmoidal ivory osteoma, Nigeria, surgical resection

## Abstract

**Background:**

Unlike small and medium size fronto-ethmoidal osteomas which are amenable to surgical excision through limited craniofacial openings, giant lesions require extensive and complex craniofacial dissection, and post lesionectomy reconstruction using an array of modern-day surgical adjuncts. This is a report of our surgical technique for the successful and esthetically fair operative resection of a giant fronto-ethmoidal osteoma in a difficult practice setting.

**Case Description:**

A 32-year-old Nigerian lady harbored a giant complex fronto-ethmoidal ivory osteoma. Deploying our understanding of modern-day advanced microsurgical anatomy and technique of skull base surgery, but under severe resource limitations, a radical total surgical resection was performed and an esthetically fair post lesionectomy reconstruction was achieved. The patient remains tumor-free in 20 months, so far, of postoperative follow-up.

**Conclusions:**

Even under severe resource limitations, inventive adaptations of modern-day skull base surgery techniques can facilitate hitherto unusual functional and esthetically successful resection of giant osteomas of the fronto-ethmoidal sinus complex.

## INTRODUCTION

Osteomas are benign usually slow-growing osseous-fibrous neoplasm. They are quite infrequent in the paranasal air sinuses and their occurrence in these locations has been put at 0.43% in one early plain sinus radiography series and 3% in a more recent sinus computed tomography survey.[6,12] The frontal-ethmoidal sinus is the most frequent site in the paranasal sinuses and even so majority of these are micro lesions averaging about 5 mm in size and hence are usually asymptomatic.[3,5,12,14] Larger lesions, those up to 30 mm in diameter, are still more infrequent, but are more wont to be symptomatic by causing varying nasal/paranasal sinus inflammatory/infective and obstructive symptoms, and occasional intracranial/orbital complications.[2,4,6,7,13,14,17] Surgical resection is then called for and many of these less than 30 mm lesions are safely excised via sundry minimally invasive techniques including external fronto-ethmoidectomy and even endoscopic endonasal methods.[10,25]

Giant fronto-ethmoidal osteomas, lesions larger than 60 mm, are very rare indeed;[16,21] they usually have wide based attachments to craniofacial bones, and extensive involvement of the anterior skull base and the orbital-nasal complex. Surgical excision of such lesions is therefore more engaging, usually involves extensive craniofacial soft tissue and bony dissection and post lesionectomy reconstruction of the surgical dissection field with modern-day advanced microsurgical tools and techniques.[16,23] We recently encountered such a giant lesion, an 80 mm fronto-ethmoidal ivory osteoma, in a developing country resource limited practice setting and here present our technique for its successful functional and esthetically fair surgical resection.

## CLINICAL AND SURGICAL DESCRIPTION OF THE CASE

**Figure 1:**
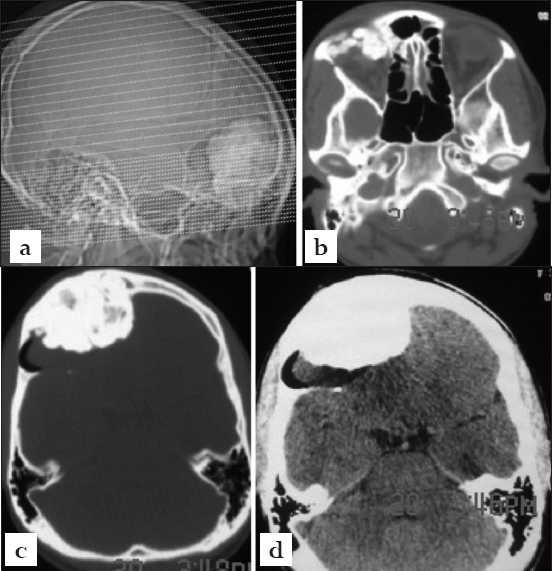
Preoperative imaging, cranial CT scanning. (a) CT scanogram showing the huge highly calcified frontal-ethmoidal mass with a cresentic radiolucent mass (mucoceles) capping its posterior rim (b) the mass involved the right orbital superior rim and roof (c) the mass is associated with expansion of the adjoining diploe suggesting the differential of intradiploic dermoid and (d) there is marked compression of the frontal lobes especially on the right

A 32-year-old female primary school teacher presented in our clinic in 2009 with a two-year-history of recurrent generalized tonic-clonic seizures. There was a prior medical history, nine years previously, of surgical excision of a frontal extracranial mass lesion. The details of this surgical procedure and the histologic findings on the excised mass were not available for our review. Clinical and neurologic examinations revealed anosmia and a healed surgical incision below the hair line in the right frontal region. There were no other neurological deficits. Visual function and ocular mobility were preserved. Cranial computed tomography, CT, scanning showed a huge 8 cm right fronto-orbital mass lesion [Figures 1a and b]. The mass was highly calcified, appeared to be expanding the diploe at its periphery and also caused marked extra-axial compression of the frontal lobes [Figures 1c and d]. There were associated isodense cystic components to the mass where it bordered the brain in keeping with mucoceles [Figure 1d]. A clinical differential diagnosis of osteoma, fibrous dysplasia, intradiploic dermoid or calcified/hyperostotic meningioma was made and surgical excision was scheduled.

### Surgical Technique

**Figure 2:**
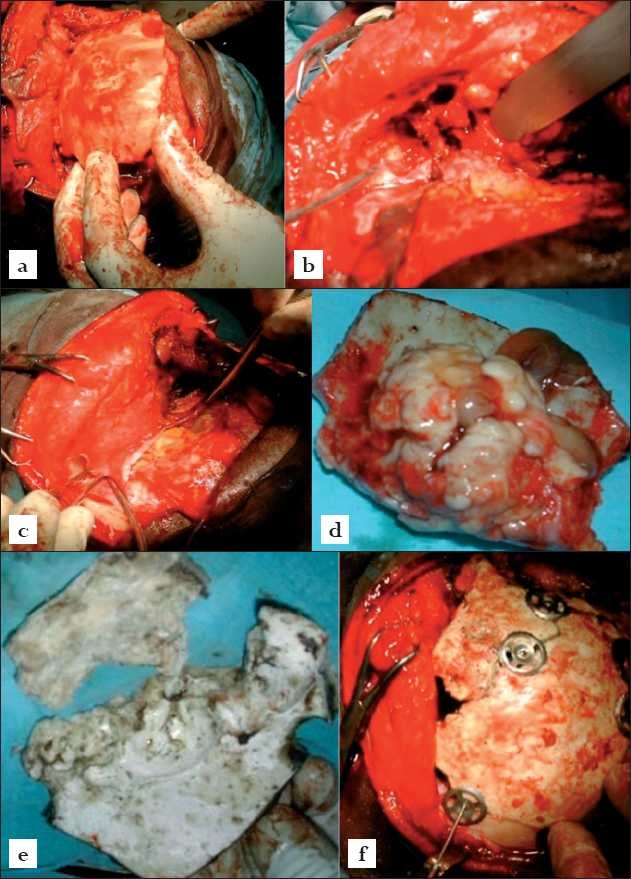
Intraoperative dissection, lesionectomy and reconstruction. (a)The cranio-orbital-nasal bone flap being raised (b) the right orbit unroofed (orbital contents retracted) and intracranial extradural fronto-ethmoidectomy achieved (c) pedicled generous pericranial flap being layered on the frontal fossa floor and (d, e) the ivory osteoma shaved off the frontal-orbital-nasal bone flap and the bone flap broken pieces after autoclaving (e) frontal-orbital-nasal calvarial rigid reconstruction being facilitated with titanium skull clamps, CranioFix^R^ (B Braun, Aesculap, Germany)

The patient was positioned supine under general endotracheal anesthesia. A bicoronal scalp flap was raised with a preauricular skin incision reaching from just above the right zygomatic process to the contralateral superior temporal line. This incision was developed from behind the hair-line, separate from the previous healed below-the-hair-line frontal incision. Next a wide-base pericranial flap was developed pedicled distally. Using the Hudson brace and Cushing’s bone perforators, strategic burr-holes were placed to raise, using the Gigli saws, a cranio-orbito-nasal bone flap [Figures 2a and b]. This craniofacial bone-flap technique spares the lateral 2/3 of the orbital rims. Disimpaction of the highly sclerotic bone flap at the naso-orbital roof was facilitated with generous use of the osteotome and mallet. Further bone resection/dissection was then carried out with the Leksell’s bone nibblers. The osteosclerotic bone flap thus raised, revealing a globular extradural multilobulated 8 cm petrous lesion from the inner table of the fronto-orbital-nasal skull bone [Figure 2d]. There were mucocoeles involving both the frontal and the anterior ethmoidal sinuses, and marked extradural compression of the frontal lobes. The dura was not breached. The diseased frontal and ethmoidal sinuses were exenterated and cranialized using bone nibblers. The sinus mucosae were thus stripped clean and intracranial extradural fronto-ethmoidectomy was achieved. So also was the right orbit unroofed and the right orbital contents decompressed [Figure 2b]. The fronto-basal soft tissue reconstruction was next effected using the pedicled pericranial flap [Figure 2c]. The latter was layered, as shown in the figure, over the floor of the frontal cranial fossa thereby sealing the remains of the fronto-ethmoidal sinuses. No further bony reconstruction of the frontal fossa floor was deemed necessary.

Attention then shifted to rigid reconstruction of the cranial-orbital-nasal opening. The ivory osteoma was shaved off the cranial flap using osteotome and mallet to save as much membraneous convexital skull bone as possible [Figures 2d and e]. The bone flaps thus retrieved were then autoclaved and used to reconstruct the calvarium [Figure 2f]. This phase of the procedure was greatly facilitated by the use of the titanium skull clamp, CranioFix^R^ (B Braun, Aesculap, Germany). The histology of the surgical specimen, reviewed by us with the pathologists, was reported as a benign bony lesion composed of trabecullae of lamellar bone and amorphous bony tissue, features of an ivory osteoma.

**Figure 3:**
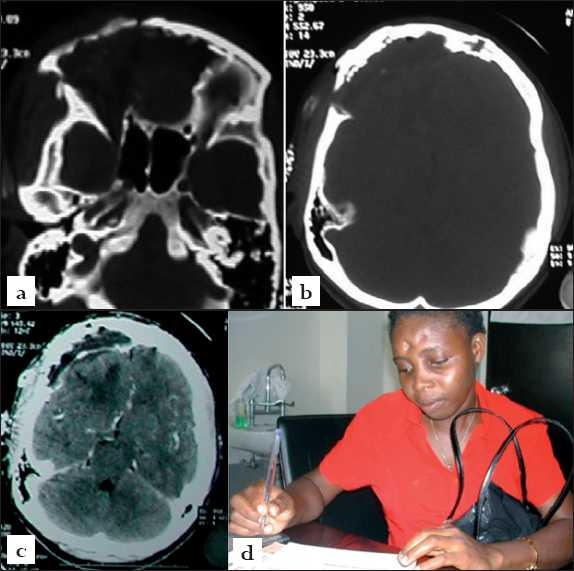
Postoperative images (a, b) Axial bone window images of the immediate postoperative cranial CT scanning confirming the operative complete lesionectomy, frontoethmoidectomy, unroofing of the right orbit and the reconstructed right orbital rim and the frontal basal skull convexity (c) good cerebral hemispheric decompression and (d) clinical picture of the patient 14 months postop

The postoperative cranial CT scanning confirmed total excision of the mass, unroofing of the right orbit and good frontobasal skull calvarial reconstruction. There was also good decompression of the frontal cerebral hemispheres [Figures 3a–c]. The patient’s postoperative clinical course was uneventful. No new post-operative neurological deficit was incurred apart from the pre-operative anosmia. She was discharged home on the postoperative day 7 and has been recurrence free in 20 months of outpatient follow up [Figure 3d]. She is still being followed up in our outpatient clinic.

## DISCUSSION

Here we present our technique for the successful surgical resection of a giant and complex fronto-ethmoidal ivory osteoma using inventive modifications of some of the modern-day skull base surgery tenets in an otherwise difficult surgical practice.

Frontal osteomas are the most common of the paranasal sinuses osteomas and are either exostotic or enostotic.[14,19] The exostotic lesions are usually bony exophytic outgrowths from the outer table of the skull presenting subcutaneously. These are usually easily shaved off the skull *via* limited scalp incisions.[16]

Enostotic frontal-ethmoidal osteomas on the other hand grow from either the outer or inner table of the skull into the respective sinuses or simply intracranial.[14] They are usually micro lesions hardly growing up to 30 mm in diameter and are also mainly excisable *via* such limited surgical openings like the supraciliary exposure, external frontoethmoidal approach, osteoplastic frontal sinusectomy or even endonasal endoscopic resection.[8,11,20] Large lesions, >30 mm in diameter, may not however be realistically amenable to total and radical resection through these restrictive openings.[10,12,16,23,25]

Ultra-large lesions, or giant frontal-ethmoidal osteomas, are those larger than 60 mm in diameter and reports of such mammoth size lesions are rare in the literature.[1,7,21,24] The lesion in our case was actually more than 80 mm large. Apart from the problem of their large size, these lesions are wont to have a wide-based attachment at the cranial fossa floor and also have infiltrative involvement of many skull base cranial-nasal-orbital structures. Only wide craniofacial dissections could therefore achieve adequate exposures and hence radical resections in such cases.[10,25] The current apparently established surgical paradigm for the latter involves raising a bicoronal scalp flap to expose the craniofacial skeleton.[9,23,28] After this, a cranial frontal bone flap is raised and other craniofacial osteotomies of varying degrees of complexities are made; radical lesionectomy is achieved, and complex craniofacial architectural reconstruction is performed to achieve post operative acceptable functional and esthetic outcome. These complex skull base surgical dissections and reconstructions are greatly facilitated with an array of modern-day surgical technological adjuncts like powered drills; specialized dissection tools like ultrasonic drills, and even CO_2_ laser; many cutting-edge technology engineered craniofacial rigid reconstruction substitutes, and miniplates and screws.[13,15,16,18,28]

Many, if not all, of these enviable resources are pretty rare luxuries in our practice. What we have going for us is only a continually-challenged inventiveness to adapt modern-day practice to our resource limitations.

In this case for instance, a single cranial-nasal-orbital bone flap using adaptive deployment of the Gigli saw, osteotome and mallet to circumscribe the limits and attachments of this huge lesion was found more practical for our situation. In the same light because our only realistic option for rigid reconstruction of the post lesionectomy defect was the patient’s own cranial bone, we salvaged as much calvarial convexital bone as possible from the patient’s tumor attached cranial flap for the same-sitting cranioplasty. Previous reports of surgical resection of giant fronto-ethmoidal osteomas in similar practice as ours show patients having to leave with unsightly post lesionectomy cranial defects that were still awaiting cranioplasty for as long as two years to the time of some of the reports.[21,24]

Obviously, one main drawback of using this tumor-attached bone for this cranioplasty is the risk of recurrence. And many cases of recurrence of frontal osteomas have indeed been reported in the literature especially after not-so-radical surgery.[22,27,28] In our own case, we have made use of our advanced skull base microsurgical training to achieve as thoroughly radical resection as possible of the lesion. We have also further tried to mitigate this risk by autoclaving the bone flap knowing that such has been known to sterilize tumor-infiltrated cranial bone flaps even when by neoplasms more aggressive than the actually benign one in our case.[26] So far, our patient remains tumor-free during a 20-month follow-up and she is still under our observation for possible recurrence.

## CONCLUSIONS

Giant fronto-ethmoidal osteomas, lesions >60 mm, are uncommon. Reports of successful functional and esthetically acceptable surgical resection of such lesions in resource-limited practices are very rare indeed. One such experience has been detailed in this paper.

## References

[1] Ataman M, Ayas K, Gursel B (1993). Giant osteoma of the frontal sinus. Rhinology.

[2] Bartlett JR (1971). Intracranial neurological complications of frontal and ethmoidal osteomas. Br J Surg.

[3] Bourdial J (1972). Frontal sinus and ethmoidofrontal sinus osteomas. Surgical indications and treatment by controlled abrasion using a drill. Ann Otolaryngol Chir Cervicofac.

[4] Bourgeois P, Fichten A, Louis E, Vincent C, Pertuzon B, Assaker R (2002). Frontal sinus osteomas: neuro-ophthalmological complications. Neurochirurgie.

[5] Brown LG (1930). Osteoma of the Frontal Sinus. Operation for removal. Proc R Soc Med.

[6] Brunori A, de Santis S, Bruni P, Delitala A, Giuffre R (1996). Chiappetta F Life threatening intracranial complications of frontal sinus osteomas: report of two cases. Acta Neurochir (Wien).

[7] Bushan B, Watal G, Ahmed A, Saxena R, Goswami K, Pathania AG (1987). Giant ivory osteoma of frontal sinus. Australas Radiol.

[8] Castelnuovo P, Giovannetti F, Bignami M, Ungari C, Iannetti G (2009). Open surgery versus endoscopic surgery in benign neoplasm involving the frontal sinus. J Craniofac Surg.

[9] Chang SC, Chen PK, Chen YR, Chang CN (1997). Treatment of frontal sinus osteoma using a craniofacial approach. Ann Plast Surg.

[10] Chiu AG, Schipor I, Cohen NA, Kennedy DW, Palmer JN (2005). Surgical decisions in the management of frontal sinus osteomas. Am J Rhinol.

[11] De Chalain T, Tan B (2003). Ivory osteoma of the craniofacial skeleton. J Craniofac Surg.

[12] Earwaker J (1993). Paranasal sinus osteomas: A review of 46 cases. Skeletal Radiol.

[13] Gutenberg A, Larsen J, Rohde V (2009). Frontal sinus osteoma complicated by extended intracranial mucocele and cerebral abscess: Neurosurgical strategy of a rare clinical entity. Cen Eur Neurosurg.

[14] Haddad FS, Haddad GF, Zaatari G (1997). Cranial osteomas: Their classification and management. Report on a giant osteoma and review of the literature. Surg Neurol.

[15] Hayden MG, Guzman R, Dulai MS, Mobley BC, Edwards MS (2009). Recurring osteoma within a calcium phosphate bone cement cranioplasty: Case report. Neurosurgery.

[16] Izci Y (2005). Management of the large cranial osteoma: Experience with 13 adult patients. Acta Neurochir (Wien).

[17] Jurlina M, Janjanin S, Melada A, Prstacic R, Veselic AS (2010). Large intracranial intradural mucocele as a complication of frontal sinus osteoma. J Craniofac Surg.

[18] Kronenberg J, Kessler A, Leventon G (1986). Removal of a frontal sinus osteoma using the CO2 laser. Ear Nose Throat J.

[19] McHugh JB, Mukherji SK, Lucas DR (2009). Sino-orbital osteoma: A clinicopathologic study of 45 surgically treated cases with emphasis on tumors with osteoblastoma-like features. Arch Pathol Lab Med.

[20] Namdar I, Edelstein DR, Huo J, Lazar A, Kimmelman CP, Soletic R (1998). Management of osteomas of the paranasal sinuses. Am J Rhinol.

[21] Olumide AA, Fajemisin AA, Adeloye A (1975). Osteoma of the ethmofrontal sinus. Case report. J Neurosurg.

[22] Panagiotopoulos V, Tzortzidis F, Partheni M, Iliadis H, Fratzoglou M (2005). Giant osteoma of the frontoethmoidal sinus associated with two cerebral abscesses. Br J Oral Maxillofac Surg.

[23] Savastano M, Guarda-Nardini L, Marioni G, Staffieri A (2007). The bicoronal approach for the treatment of a large frontal sinus osteoma. A technical note. Am J Otolaryngol.

[24] Shehu BB, Zaman JN (2001). Giant Osteoma of the Frontoethmoidal sinus: A Case Report. Niger J Surg Res.

[25] Strek P, Zagolski O, Skladzien J, Kurzynski M, Dyduch G (2007). Osteomas of the paranasal sinuses: Surgical treatment options. Med Sci Monit.

[26] Vanaclocha V, Sfiiz-Sapena N, Garcia-Casasola C, De Alava E (1997). Cranioplasty with autogenous autoclaved calvarial bone flap in the cases of tumoural invasion. Acta Neurochir (Wien).

[27] Vonofakos DA, Karakoulakis E (1981). Recurrent osteoma overlying cranioplasty. Case report. J Neurosurg.

[28] Wanyura H, Kaminski A, Stopa Z (2005). Treatment of osteomas located between the anterior cranial base and the face. J Craniomaxillofac Surg.

